# Traditional, Complementary, and Integrative Medicine on X (Formerly Twitter) From 2015 to 2024 Across English, Spanish, and French: Retrospective Observational Study

**DOI:** 10.2196/97760

**Published:** 2026-07-27

**Authors:** Jahinna Duplaix, Sébastien Abad, Marion Bruna, Arnaud Legout, Alexis Nommer, Deborah Nourrit-Lucas, Grégory Ninot

**Affiliations:** 1 Institut Desbrest d'Épidémiologie et de Santé Publique, UMR 1318 Université de Montpellier – INSERM Montpellier, Occitanie France; 2 Centre Inria d'Université Côte d'Azur Valbonne, Provence-Alpes-Côte d'Azur France; 3 Institut Regional du Cancer de Montpellier Montpellier, Occitanie France; 4 Institut Universitaire de France Paris, Île-de-France France

**Keywords:** social media, trend analysis, X, Twitter, traditional, complementary, and integrative medicine, supportive care, infoveillance, health information, infodemiology

## Abstract

**Background:**

Social media platforms have become important spaces for the circulation and discussion of health information. X (formerly Twitter) is one such space where traditional, complementary, and integrative medicine (TCIM)–related terms circulate in public health discourse. However, longitudinal TCIM-related mention patterns on social media, particularly during the COVID-19 pandemic period, remain poorly documented.

**Objective:**

The study aimed to characterize temporal mention patterns of TCIM-related terms on X across English, Spanish, and French between 2015 and 2024, including the COVID-19 pandemic period.

**Methods:**

We conducted a retrospective observational infodemiology study using raw, nonnormalized annual mention counts from Brandwatch-indexed public X content in English, Spanish, and French that contained 39 predefined TCIM-related terms from 2015 to 2024. Descriptive analyses summarized cumulative mentions and annual mention counts. Exploratory trend analyses were restricted to the 2015 to 2019 prepandemic period. For each term-language combination, ordinary least squares regression was fitted with annual mention count as the dependent variable and calendar year as the independent variable. Exploratory linear trend signals were retained when *R*^2^≥0.70 and an exact permutation test on the slope yielded *P*≤.05 (120 permutations).

**Results:**

Cumulative mentions of the 39 TCIM-related terms totaled 191,994,848 over 2015 to 2024. The most frequently mentioned terms were “yoga” (79,618,183), “meditation” (55,560,372), and “mindfulness” (18,915,377), together accounting for 80.3% (154,093,932/191,994,848) of all mentions. Raw cumulative mentions were predominantly indexed in English (170,100,867/191,994,848, 88.6%) compared with Spanish (17,171,541/191,994,848, 8.9%) and French (4,722,440/191,994,848, 2.5%). Between 2019 and 2020, “meditation” showed the largest absolute increase in raw mention counts (1,935,949/3,627,557, 53.4%), whereas “ayurveda” showed the largest relative increase (400,624/214,901, 186.4%). Exploratory 2015 to 2019 trend analyses identified 14 linear trend signals in English (n=13, 92.9% decreasing; n=1, 7.1% increasing: “intermittent fasting,” 161,584/70,496, 229%), 11 in French (n=5, 45.5% increasing), and 8 in Spanish (3 increasing). “Reiki” and “reflexology” decreased in all 3 languages.

**Conclusions:**

Between 2015 and 2024, raw TCIM-related mention counts on X were highly concentrated in a small set of wellness and mind-body labels and were predominantly in English compared with Spanish and French. No sustained aggregate surge was evident in the raw descriptive data during the COVID-19 pandemic period, although some terms such as “ayurveda” showed short-term fluctuations. These findings should be interpreted as platform-specific raw mention patterns, not as evidence of normalized public interest, sentiment, user attitudes, professional practice, or actual TCIM use.

## Introduction

Traditional, complementary, and integrative medicine (TCIM) has received considerable media attention over the past decade [1,2]. Social media platforms, particularly X (formerly Twitter), have played a central role in disseminating health-related information and discourse [3]. X is a platform for rapid microblogging shaped by virality mechanisms (hashtags, retweets, and algorithmic amplification). On this platform, influencers and microcommunities can contribute to the amplification of TCIM-related health discourse and polarization during crises [4].

The COVID-19 pandemic has been associated with increased visibility of TCIM-related discussions and misinformation on social media, alongside temporal trends and variations in discourse tone [2]. The COVID-19 pandemic period also contributed to consolidating the concept of an infodemic [5]. Lockdowns in many English-, Spanish-, and French-speaking countries may have increased reliance on digital technologies and social media for health information, protective measures, and perceived responses to the virus, particularly in contexts of uncertainty and constrained health care resources [6,7]. X has been widely studied in relation to the rapid spread of controversial content and urgent health information seeking [8]. Whether the period from 2015 to 2024 was marked by a gradual rise, a possible COVID-19 (2020 to 2021) surge, or a postpandemic stabilization (2022 to 2024) in TCIM-related mention patterns on X remains an open empirical question.

Researchers and health organizations emphasize that the umbrella term TCIM covers a wide range of heterogeneous meanings. A TCIM-related term can refer to a broad system of medicine, an approach, a discipline, a technique, or a specific remedy-related label. For example, the lexicon may include broad labels such as traditional Chinese medicine, discipline labels such as acupuncture, technique labels such as moxibustion, or specific remedy-related labels such as St John’s wort. The meaning of each term may vary depending on context and may overlap with supportive care, self-care, traditional medicine, complementary therapies, lifestyle practices, or spiritual practices [9,10].

Furthermore, the uses, representations, and forms of legitimation of TCIM vary across national contexts and language-specific semantics. A traditional medicine in one country can be an officially recognized medicine in another. Researchers have described this variation across health care systems and contexts, with marked differences in prevalence, use, and regulation [11-13]. On social media platforms, language structures communities, interpretive frameworks, and dissemination dynamics. Language is not simply a vector of communication but also a marker of specific information cultures [14]. Analyses from computational sociolinguistics applied to X indicate that, although more than a hundred languages are used on the platform, textual production remains highly concentrated. English consistently ranks first and accounts for a substantial proportion of messages, while Spanish is consistently among the most widely used languages worldwide, in line with the widespread geographical distribution of its linguistic communities [15,16]. Although French does not reach the level of the dominant languages, it nevertheless appears repeatedly among the major languages on the platform. Its transnational presence, particularly in Europe, French-speaking Africa, and North America, may contribute to its relatively high visibility compared with languages that are more confined to national spaces [16]. Most languages present on the platform are part of a long list characterized by low overall volumes, although some may occupy a central place in specific local contexts; these hierarchies remain sensitive to observation periods, language detection methods, and sampling strategies, particularly when based on geolocated data [15]. The multilingual approach adopted in this study aimed to reduce the Anglocentrism bias, given that English is a historically, socially, and technically dominant language in the digital realm [17].

Three research questions guided this study: (1) whether raw TCIM-related mention counts on X increased over time, with a possible COVID-19 pandemic–period surge; (2) how cumulative raw mentions were distributed across the predefined semantic levels of approaches, disciplines, and techniques; and (3) whether temporal patterns of mentions differed across English, Spanish, and French over the decade.

## Methods

### Principle

We conducted a retrospective observational infodemiology study using annual TCIM-related mention counts from public content posted on X [18]. Word- and phrase-level time series from X have been used in previous health research and infodemiology studies [19,20]. We analyzed aggregated annual mention counts for public X posts containing at least one term from the study lexicon; no participants were recruited. The observation period ran from January 2015 to December 2024 and was used to document decade-long raw mention patterns, including a global pandemic episode (COVID-19 pandemic), during which TCIM-related discussions and misinformation on social media have been reported [2].

### Lexicon Construction and Multilingual Strategy

The lexicon aimed to cover TCIM-related mentions at 3 semantic levels: approaches, disciplines, and techniques, with remedy-related labels treated as part of the technique level when relevant. This strategy was intended to reduce omissions caused by differences in terminology across communities and discourse contexts [21]. The lexicon was compiled from three sources: (1) institutional references, including World Health Organization (WHO) reports on TCIM [22] and information sheets from the French Ministry of Health [23], to frame the scope of TCIM; (2) encyclopedic references from Wikipedia, which is often used in health-related text mining as a source of vocabulary and lexical variants [24]; and (3) triangulation with 3 generative AI tools, DeepSeek, ChatGPT (OpenAI), and Microsoft Copilot, used as controlled brainstorming tools to propose candidate terms. The use of generative models to assist in refining search strategies and search terms has been discussed in recent literature [25,26]. Triangulation was used to improve lexical coverage and reduce dependence on a single source [27].

The exact prompt used for this step is provided in Textbox S1 in Multimedia Appendix 1. It was executed independently for each AI tool on April 21, 2025. The prompt focused on terms likely to be written, searched for, used as hashtags, or discussed on X (formerly Twitter). It prioritized terms that were widely recognized in public discourse, likely to generate substantial mention volumes, and relevant to TCIM. It excluded names of people, brands, clinics, commercial products, and biomedical treatments not generally addressed within the TCIM field. The prompt also specified that AI-generated outputs should not be interpreted as evidence of clinical effectiveness, public health relevance, frequency of use, or real-world use. The AI-assisted step identified 104 candidate TCIM-related terms in at least one AI tool, including 39 terms identified by all 3 AI tools (Table S1 in Multimedia Appendix 1). AI outputs were used only to generate candidate terms; final inclusion in the main lexicon required review by the authors and consistency with the study scope.

In total, the study retained 39 English seed terms: 10 (25.6%) for approaches, 16 (41%) for disciplines, and 13 (33.3%) for techniques. The retained terms are “yoga,” “meditation,” “mindfulness,” “ayurveda,” “essential oils,” “homeopathy,” “reiki,” “chiropractic,” “tai chi,” “acupuncture,” “intermittent fasting,” “traditional medicine,” “hypnotherapy,” “music therapy,” “ketogenic diet,” “kinesiology,” “reflexology,” “art therapy,” “acupressure,” “phytotherapy,” “qi gong,” “shiatsu,” “traditional Chinese medicine,” “osteopathy,” “naturopathy,” “shamanism,” “energy healing,” “EMDR (eye movement desensitization and reprocessing),” “sophrology,” “integrative medicine,” “forest bathing,” “neurofeedback,” “energy medicine,” “complementary medicine,” “St John’s wort,” “moxibustion,” “thermal therapy,” “Buteyko,” “anthroposophic medicine.”

These English seed terms were translated into Spanish and French to reduce Anglocentric bias and improve coverage across 3 widely represented languages on X [17], without assuming perfect semantic equivalence across languages. Relevant lexical variants were included when applicable. The 39 English terms, their Spanish and French translations, and the lexical variants used in the searches are provided in Table S2 in Multimedia Appendix 1.

### Database

Data were obtained from Brandwatch Consumer Research (Cision Ltd), a social listening platform that supports historical queries and aggregated exports. Brandwatch was selected because it provides large-scale historical coverage, standardized queries, and has been used in previous academic work on social data mining in health [28,29]. Compared with partial API feeds, provider-based access can reduce some coverage constraints associated with sampled data streams [30]. In Brandwatch terminology, a “mention” corresponds to a content item returned by a query. According to Brandwatch documentation, Brandwatch is an official X partner and indexes public posts as mentions, including reposts, retweets, and quote posts [31]. The study team received an Excel spreadsheet containing aggregated annual mention counts. The primary analytic variable was the raw, nonnormalized annual mention count for each term, stratified by language: English, Spanish, and French. Language classification was provided in the Brandwatch export and was not manually assigned post hoc by the study team. Because only aggregate exports were available, postlevel validation of language classification, including code-switching errors, was not possible. No annual denominator, such as total X activity, language-specific posting volume, or active users by language, was available.

### Ethical Considerations

This study analyzed provider-generated aggregate counts derived from publicly available X content. The study team did not access individual-level posts, usernames, profile information, or other directly identifiable personal data. No users were contacted, recruited, or interacted with. Because the analyses were restricted to nonidentifiable aggregate data by year, term, and language, informed consent was not required. Data handling and reporting followed applicable ethical and privacy principles for internet research [32].

### Statistical Analysis

All analyses were based on raw, nonnormalized annual mention counts. Descriptive analyses were conducted in 2 complementary ways. First, for each language, mention counts were aggregated across the 2015 to 2024 period to summarize the overall distribution of TCIM-related terms within each language-specific corpus. Second, annual mention counts were aggregated across English, Spanish, and French to examine temporal patterns in the predefined TCIM-related corpus. These descriptive results were reported using tables and figures, in accordance with infodemiology approaches to time-series reporting [18].

Exploratory trend analyses were restricted to the 2015 to 2019 prepandemic window to avoid fitting a single linear trend across the full 2015 to 2024 period, which included the COVID-19 pandemic period and may have involved a structural break [33]. The complete 2015 to 2024 series was retained for descriptive reporting. For each of the 39 terms within each language, an ordinary least squares linear regression was fitted with annual mention count as the dependent variable and calendar year as the independent variable. Because only 5 annual observations were available and multiple term-language comparisons were performed, these analyses were treated as exploratory and hypothesis-generating rather than confirmatory.

Two prespecified filters were applied to identify exploratory linear trend signals. First, goodness of linear fit was assessed using the coefficient of determination (*R*^2^) and only terms with *R*^2^≥0.70 were retained. Second, the slope was evaluated using an exact permutation test, enumerating all 5!=120 permutations of the 5 annual values across years. The 2-sided *P* value was calculated as the proportion of permuted slopes at least as extreme as the observed slope. Terms were retained as showing an exploratory linear trend signal when both criteria were met: *R*^2^≥0.70 and *P*≤.05. No multiplicity adjustment was applied because these analyses were designed to identify exploratory signals rather than provide confirmatory evidence.

For retained terms, changes from 2015 to 2019 were summarized using the estimated slope and relative change. Uncertainty around 2015 to 2019 relative changes was summarized using 95% bootstrap CIs obtained by nonparametric resampling with replacement and the percentile method, using the 2.5th and 97.5th percentiles [34]. Bootstrap CIs are reported in Figures S1-S3 in Multimedia Appendix 3.

## Results

Across the 39 predefined TCIM-related terms, cumulative raw mention counts on X were highly concentrated in a small number of terms (Table 1). Overall, the most frequently mentioned terms were yoga (79,618,183/191,994,848, 41.5%) and meditation (55,560,372/191,994,848, 28.9%), followed by mindfulness (18,915,377/191,994,848, 9.9%). Together, these 3 terms accounted for 154,093,932 mentions, representing 80.3% (154,093,932/191,994,848) of all cumulative raw mentions across the 39 lexicon entries. Percentages were calculated using the total cumulative raw mention count across the 39 entries as the denominator (191,994,848). Most raw cumulative mentions were indexed in English (170,100,867/191,994,848, 88.6%) compared with Spanish (17,171,541/191,994,848, 8.9%) and French (4,722,440/191,994,848, 2.5%).

**Table 1:** Cumulative raw mention counts of 39 TCIM-related terms on X, by semantic level and language, 2015 to 2024^a^.

TCIM-related lexicon entry	Semantic level^b^	English, n (%)	Spanish, n (%)	French, n (%)	Total, n^c^
Yoga	Discipline	71,203,218 (41.9)	7,370,196 (42.9)	1,044,769 (22.1)	79,618,183
Meditation	Technique	50,874,183 (29.9)	3,238,276 (18.9)	1,447,913 (30.7)	55,560,372
Mindfulness	Technique	17,881,645 (10.5)	858,169 (5)	175,563 (3.7)	18,915,377
Ayurveda	Approach	4,207,734 (2.5)	166,945 (1)	22,051 (0.5)	4,396,730
Essential oils	Technique	3,265,486 (1.9)	248,465 (1.4)	274,710 (5.8)	3,788,661
Homeopathy	Approach	1,775,122 (1)	1,350,867 (7.9)	478,647 (10.1)	3,604,636
Reiki	Discipline	2,432,571 (1.4)	860,593 (5)	106,961 (2.3)	3,400,125
Chiropractic	Discipline	3,276,148 (1.9)	67,863 (0.4)	37,149 (0.8)	3,381,160
Tai chi	Discipline	2,989,164 (1.8)	225,072 (1.3)	116,272 (2.5)	3,330,508
Acupuncture	Discipline	2,189,627 (1.3)	500,189 (2.9)	73,752 (1.6)	2,763,568
Intermittent fasting	Technique	1,727,157 (1)	540,311 (3.1)	28,842 (0.6)	2,296,310
Traditional medicine	Approach	480,835 (0.3)	336,393 (2)	53,749 (1.1)	870,977
Hypnotherapy	Discipline	832,497 (0.5)	23,836 (0.1)	7970 (0.2)	864,303
Music therapy	Discipline	649,692 (0.4)	184,165 (1.1)	13,729 (0.3)	847,586
Ketogenic diet	Technique	721,685 (0.4)	98,343 (0.6)	16,546 (0.4)	836,574
Kinesiology	Discipline	534,477 (0.3)	282,469 (1.6)	14,907 (0.3)	831,853
Reflexology	Discipline	531,612 (0.3)	69,505 (0.4)	71,425 (1.5)	672,542
Art therapy	Discipline	526,962 (0.3)	22,149 (0.1)	25,244 (0.5)	574,355
Acupressure	Technique	501,962 (0.3)	50,946 (0.3)	18,567 (0.4)	571,475
Phytotherapy	Discipline	441,289 (0.3)	62,194 (0.4)	34,100 (0.7)	537,583
Qigong	Discipline	409,247 (0.2)	58,106 (0.3)	21,950 (0.5)	489,303
Shiatsu	Discipline	345,893 (0.2)	61,902 (0.4)	34,129 (0.7)	441,924
Traditional Chinese medicine	Approach	262,371 (0.2)	117,922 (0.7)	48,926 (1)	429,219
Osteopathy	Discipline	228,747 (0.1)	87,488 (0.5)	111,469 (2.4)	427,704
Naturopathy	Approach	304,105 (0.2)	31,332 (0.2)	86,562 (1.8)	421,999
Shamanism	Approach	224,880 (0.1)	119,668 (0.7)	24,059 (0.5)	368,607
Energy healing	Discipline	324,195 (0.2)	8925 (0.1)	17,962 (0.4)	351,082
EMDR (eye movement desensitization and reprocessing)	Technique	240,630 (<0.1)	28,696 (0.2)	21,675 (0.5)	291,001
Sophrology	Discipline	7955 (<0.1)	1911 (<0.1)	226,662 (4.8)	236,528
Integrative medicine	Approach	156,768 (0.1)	30,189 (0.2)	5849 (0.1)	192,806
Forest bathing	Technique	165,892 (0.1)	455 (<0.1)	6247 (0.1)	172,594
Neurofeedback	Technique	125,184 (0.1)	25,809 (0.2)	7541 (0.2)	158,534
Energy medicine	Approach	94,899 (0.1)	2231 (<0.1)	1108 (<0.1)	98,238
Complementary medicine	Approach	60,507 (<0.1)	17,204 (0.1)	1906 (<0.1)	79,617
St John’s wort	Technique	48,772 (<0.1)	9078 (0.1)	7953 (0.2)	65,803
Moxibustion	Technique	34,061 (<0.1)	10,687 (0.1)	1294 (<0.1)	46,042
Thermal therapy	Technique	6653 (<0.1)	192 (<0.1)	23,207 (0.5)	30,052
Buteyko breathing technique	Technique	15,651 (<0.1)	958 (<0.1)	121 (<0.1)	16,730
Anthroposophic medicine	Approach	1391 (<0.1)	1842 (<0.1)	10,954 (0.2)	14,187
Total	—^d^	170,100,867	17,171,541	4,722,440	191,994,848

^a^Values are cumulative raw mention counts from 2015 to 2024 and were not normalized by total annual X activity or language-specific posting volume.

^b^Semantic level refers to the classification of each term as an approach, discipline, or technique.

^c^The total column corresponds to the sum of English, Spanish, and French mention counts.

^d^Not applicable.

When results were stratified by language, the highest cumulative counts were observed in English for the main high-volume terms. Yoga accumulated 41.9% (71,203,218/170,100,867) mentions in English compared with 42.9% (7,370,196/17,171,541) in Spanish and 22.1% (1,044,769/4,722,440) in French. Meditation accumulated 29.9% (50,874,183/170,100,867) mentions in English, compared with 18.9% (3,238,276/17,171,541) in Spanish and 30.7% (1,447,913/4,722,440) in French. In Spanish, the largest cumulative counts were observed for yoga (7,370,196/17,171,541, 42.9%) and meditation (3,238,276/17,171,541, 18.9%), followed by homeopathy (1,350,867/17,171,541, 7.9%). In French, the largest cumulative counts were for meditation (1,447,913/4,722,440, 30.7%) and yoga (1,044,769/4,722,440, 22.1%), followed by homeopathy (478,647/4,722,440, 10.1%).

Two terms contrasted with this English-dominant pattern, with cumulative mentions primarily concentrated in French: sophrology (226,662/236,528, 95.8% French vs 7955/236,528, 3.4% English and 1911/236,528, 0.8% Spanish) and thermal therapy (23,207/30,052, 77.2% in French vs 6653/30,052, 22.1% in English and 192/30,052, 0.6% in Spanish).

**Figure 1:**
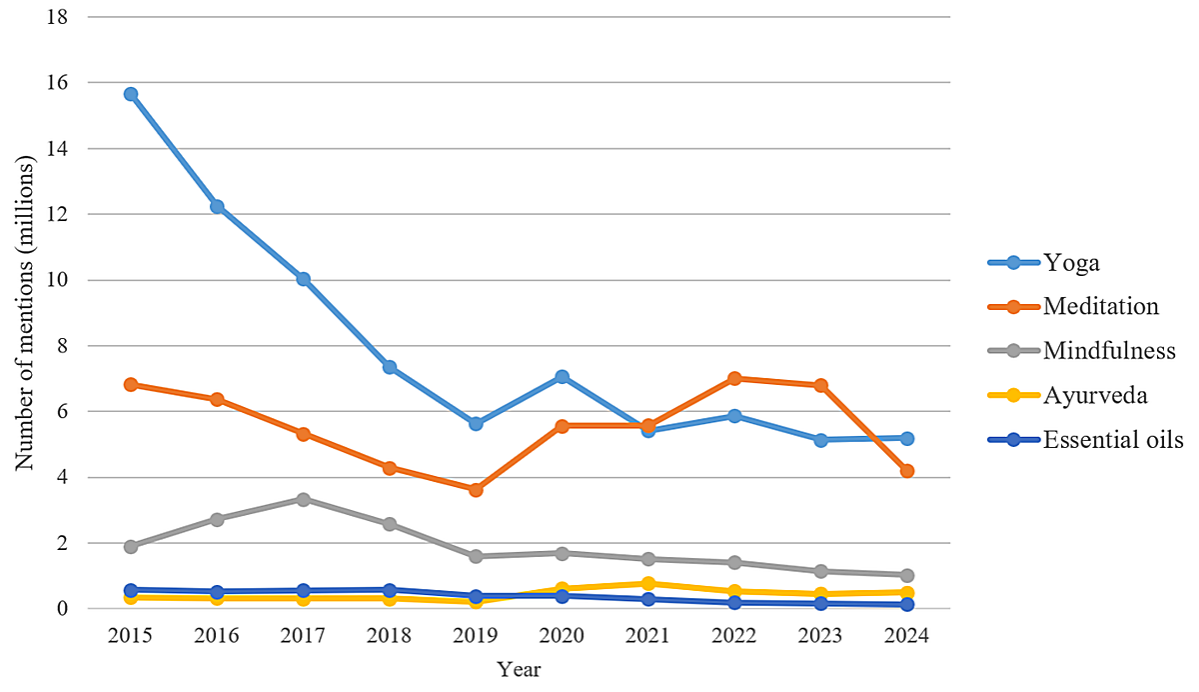
Annual raw mention counts for the 5 most frequently mentioned traditional, complementary, and integrative medicine (TCIM)–related terms on X, 2015 to 2024, pooled across English, Spanish, and French. Values are expressed in millions and were not normalized by total X activity or language-specific posting volume.

Figure 1 presents annual raw mention counts for the 5 most frequently mentioned TCIM-related terms, pooled across English, Spanish, and French. Yoga had the highest annual count throughout the period. It decreased from 15,659,632 mentions in 2015 to 5,635,251 in 2019, increased to 7,061,449 in 2020, and then varied between 5,141,235 and 5,866,108 between 2021 and 2024. Meditation decreased from 6,822,878 in 2015 to 3,627,557 in 2019, increased to 5,563,506 in 2020, reached 7,002,325 in 2022, and decreased to 4,195,184 in 2024 (Figure 1; Table S3 in Multimedia Appendix 2).

Among the remaining terms shown in Figure 1, mindfulness peaked in 2017 (3,328,490 mentions) and then decreased to 1,021,990 in 2024. Ayurveda had its lowest annual count in 2019 (214,901 mentions) and its highest annual count in 2021 (773,696 mentions). Essential oils peaked in 2018 (580,125 mentions) and decreased to 132,201 mentions in 2024. Annual raw mention counts for all 39 TCIM-related terms are provided in Table S3 in Multimedia Appendix 2.

Among other high-volume terms not displayed in Figure 1, homeopathy peaked in 2018 (691,708 mentions) and decreased to 153,688 mentions in 2024. Reiki, chiropractic, tai chi, and acupuncture reached their maximum annual counts between 2015 and 2018 and had lower counts in 2024. Between 2019 and 2020, meditation showed the largest absolute increase in raw mention counts, rising from 3,627,557 to 5,563,506 mentions (1,935,949/3,627,557, 53.4%), whereas ayurveda showed the largest relative increase, rising from 214,901 to 615,525 mentions (400,624/214,901, 186.4%; Table S3 in Multimedia Appendix 2).

**Figure 2:**
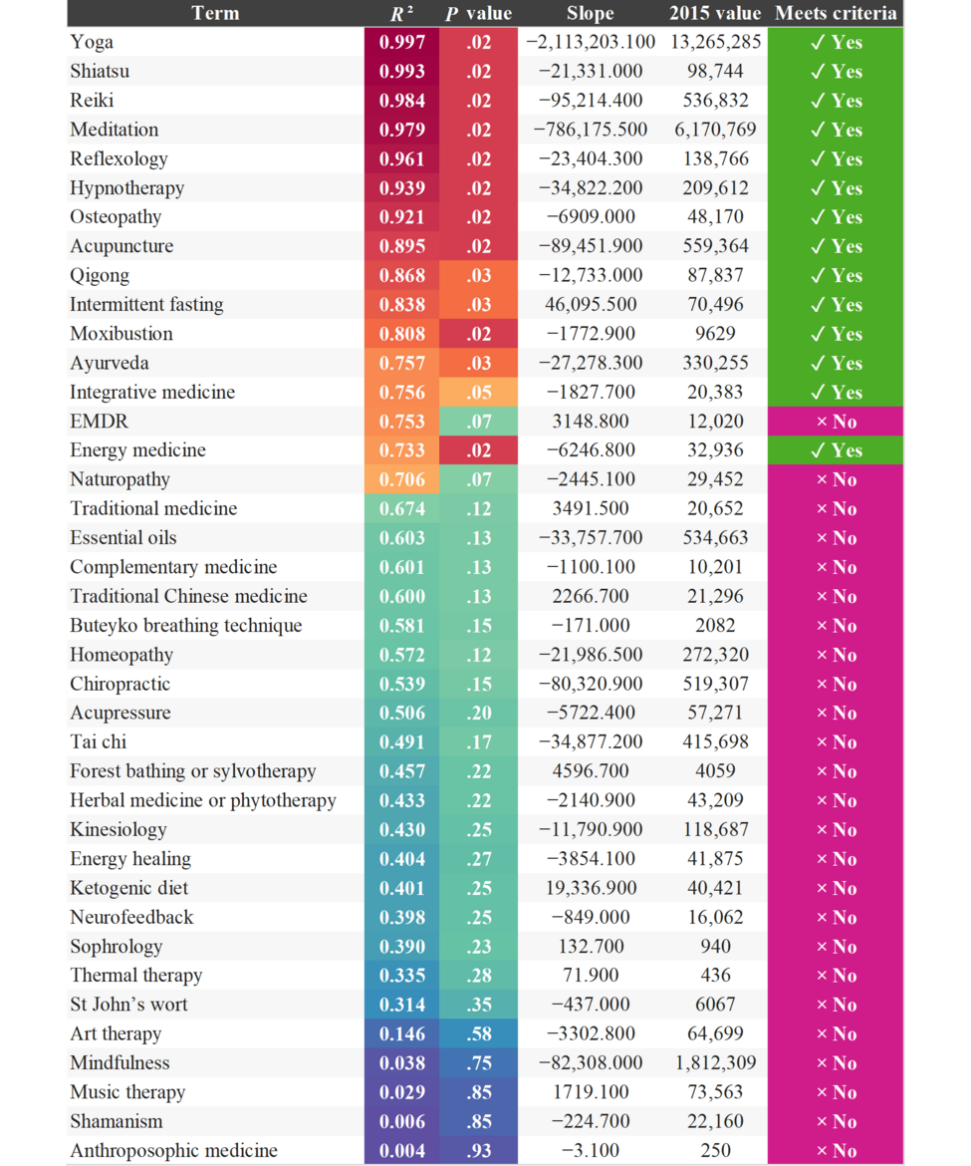
Exploratory trend analysis results of 39 English-language traditional, complementary, and integrative medicine (TCIM)–related terms on X, 2015 to 2019. The slope represents the estimated annual change in the raw number of mentions, and the 2015 value represents the raw mention count in 2015. Terms met the predefined criteria when *R*^2^≥0.70 and *P*≤.05. EMDR: eye movement desensitization and reprocessing.

Figure 2 presents the English-language exploratory trend analysis results for each of the 39 TCIM-related terms. For each term, the figure reports the coefficient of determination (*R*^2^), the *P* value from the exact permutation test on the slope, the estimated annual slope, the 2015 raw mention count, and whether the term met the predefined exploratory criteria of *R*^2^≥0.70 and *P*≤.05. The corresponding multilingual results, including French- and Spanish-language analyses, are provided in Figure S4 in Multimedia Appendix 3. Applying these exploratory criteria identified 14 English-language, 11 French-language, and 8 Spanish-language term-language combinations that met the predefined signal-detection thresholds.

Among the 14 English-language terms, 1 (7.1%) had a positive slope and 13 (92.9%) had negative slopes. Intermittent fasting was the only English-language term meeting the predefined exploratory criteria with an increasing trend signal, with an estimated annual increase of 46,095.5 raw mentions. Raw mentions increased from 70,496 in 2015 to 232,080 in 2019, corresponding to a 229% (161,584/70,496) increase. The largest negative slopes were observed for yoga (–2,113,203.1 raw mentions per year), meditation (–786,175.5), reiki (–95,214.4), acupuncture (–89,451.9), and hypnotherapy (–34,822.2).

Among the 11 French-language terms, 5 (45.5%) had positive slopes and 6 (54.5%) had negative slopes. Positive slopes were observed for homeopathy (+33,281.8 raw mentions per year), chiropractic (+2454.7), ketogenic diet (+432.8), shamanism (+281.0), and complementary medicine (+70.5). Negative slopes were observed for yoga (–19,738.0), reflexology (–5676.8), reiki (–3629.1), tai chi (–1804.3), shiatsu (–1694.0), and ayurveda (–144.5; Figure S4 in Multimedia Appendix 3).

Among the 8 Spanish-language terms, 3 had positive slopes and 5 had negative slopes. Positive slopes were observed for intermittent fasting (+6815.5 raw mentions per year), integrative medicine (+760.5), and forest bathing (+35.9). Negative slopes were observed for meditation (–63,200.0), reiki (–30,248.2), reflexology (–3015.7), neurofeedback (–514.3), and energy healing (–85.5; Figure S4 in Multimedia Appendix 3). Bootstrap percentile 95% CIs for the 2015 to 2019 relative changes are provided in Figures S1-S3 in Multimedia Appendix 3.

**Figure 3:**
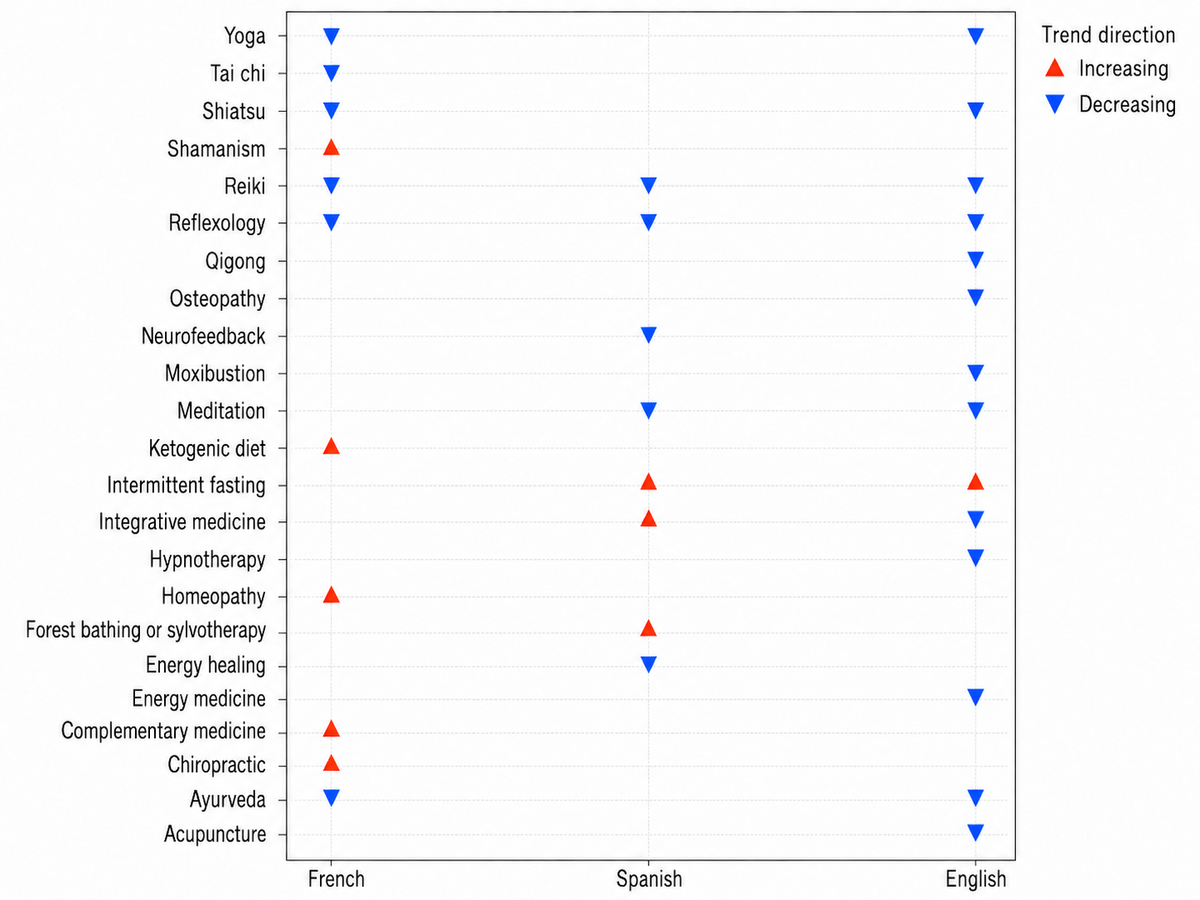
Direction of exploratory trend signals for traditional, complementary, and integrative medicine (TCIM)–related terms on X by language, 2015 to 2019. Upward-pointing triangles indicate increasing trends, and downward-pointing triangles indicate decreasing trends. Only term-language combinations meeting the predefined criteria (*R*^2^≥0.70 and *P*≤.05) are shown. The absence of a symbol indicates that the term did not meet these criteria for the corresponding language.

Figure 3 compares the direction of exploratory trend signals from 2015 to 2019 across English, Spanish, and French. Two terms met the predefined exploratory criteria in all 3 languages and showed a consistent decreasing direction: reiki and reflexology. Several terms met these criteria in 2 languages with the same direction: yoga, shiatsu, and ayurveda decreased in French and English; meditation decreased in Spanish and English; and intermittent fasting increased in Spanish and English. Integrative medicine showed divergent directions, increasing in Spanish and decreasing in English. The remaining language-specific signals were tai chi, shamanism, ketogenic diet, homeopathy, complementary medicine, and chiropractic in French; neurofeedback, forest bathing, and energy healing in Spanish; and qigong, osteopathy, moxibustion, hypnotherapy, acupuncture, and energy medicine in English.

## Discussion

### Principal Results

Across the 2015 to 2024 period, the raw, nonnormalized mention counts did not show a sustained aggregate increase in the predefined TCIM-related corpus on X. No sustained aggregate surge was evident in the raw descriptive series during the COVID-19 pandemic period, although term-level fluctuations were observed. This may partly reflect informational saturation during the pandemic [35]. Prominent topics such as vaccines, lockdowns, and pandemic-related policies dominated online health discussions on X [36,37]. However, the analysis could not assess the effect of platform-level changes on indexed mention volumes, including the 2023 rebranding and changes in governance or visibility algorithms. Year-on-year variations were mixed. Between 2019 and 2020, meditation showed the largest absolute increase in raw mention counts, rising from 3,627,557 to 5,563,506 mentions (1,935,949/3,627,557, 53.4%). Ayurveda showed the largest relative increase, rising from 214,901 to 615,525 mentions (400,624/214,901, 186.4%; Table S3 in Multimedia Appendix 2). These changes should be interpreted as isolated term-level fluctuations rather than evidence of a broader TCIM surge. They may be compatible with broader wellness and stress management vocabularies circulating online. However, the data do not allow inference about motivations, use, therapeutic intent, or endorsement. Similarly, the decrease in yoga mentions between 2015 and 2019 may reflect several nonmutually exclusive mechanisms, including lexical diversification, changes in platform activity, or shifts toward other platforms [38,39]. These mechanisms could not be tested in this study.

Over the decade, disciplines and techniques were more frequently mentioned than broad approaches within the predefined lexicon. This finding should be interpreted descriptively because the semantic groupings were heterogeneous and contained different modalities of practice: 10 approaches, 16 disciplines, and 13 techniques. High-volume labels such as yoga, meditation, and mindfulness are also culturally broad wellness terms that may dominate aggregate counts regardless of category logic. Therefore, this result should not be interpreted as evidence that disciplines or techniques are generally more visible than broad approaches across TCIM discourse overall. Rather, it indicates that, within this specific lexicon and Brandwatch-indexed X corpus, practice and wellness labels were more frequently captured than broad umbrella labels. From a monitoring perspective, this pattern suggests that a harmonized classification of evidence-based TCIM programs could improve their recognition by digital tools and their traceability within health and prevention organizations [40,41]. This aligns with a global health policy context aimed at strengthening evidence, quality, safety, and access to TCIM [42].

The predominance of English in TCIM-related mentions (170,100,867/191,994,848, 88.6%), compared with Spanish (17,171,541/191,994,848, 8.9%) and French (4,722,440/191,994,848, 2.5%), is consistent with previous literature [14]. These cross-language differences may reflect variations in platform activity, public policies, and the influence of country-specific traditional medicines [21], but these mechanisms were not directly tested. Consequently, mention frequency in each language should not be interpreted as evidence of the views, attitudes, or practices of the corresponding population. Language should be considered a proxy for linguistic space, not a direct proxy for culture, nationality, or comparable discourse communities. Translated terms may also differ in meaning and use across languages.

A central issue for interpretation is the heterogeneity of the TCIM umbrella concept. The predefined lexicon included broad systems of medicine, lifestyle and wellness labels, body-mind practices, diet-related terms, clinically framed interventions, and contested modalities. Therefore, the aggregate corpus should not be interpreted as a homogeneous measure of TCIM discourse. The concentration of 80.3% (154,093,932/191,994,848) of cumulative mentions in yoga, meditation, and mindfulness indicates the dominance of wellness and body-mind vocabularies within the selected lexicon. It does not imply that all TCIM domains were equally visible or that these high-volume terms were always used in explicitly medical, therapeutic, or TCIM-related contexts. In a geopolitical period marked by war, infectious disease, misinformation, climate change, occupational pressure, and resource constraints, this predominance may align with broader online wellness and self-care vocabularies, particularly those related to stress management and mindfulness [7]. More broadly, these patterns may be discussed in relation to online interest in nonpharmacologic or complementary options and concerns about trust in pharmaceutical manufacturers, although existing evidence remains population- and context-specific, including cardiovascular-risk populations [43]. However, the present data cannot show that users turned toward approaches perceived as more “natural,” or whether posts reflected mistrust, criticism, or patient empowerment. These findings suggest that social media constitute an important space for tracking the circulation of TCIM-related health vocabularies [20]. Further qualitative, sentiment, and user-level analyses would be needed to examine therapeutic preferences, stress management narratives, and broader relationships to conventional medicine.

### Limitations

The study measured the frequency of predefined TCIM-related mentions on X, not actual practice use, sentiment, or user intent. Mentions may reflect positive, neutral, or negative opinions, as well as promotion, criticism, controversy, humor, media coverage, advertising, or reposting. Without sentiment, contextual, or user-level analyses, changes in volume cannot be interpreted as evidence of endorsement, rejection, or real-world trends in TCIM practices. This limitation is widely recognized in Twitter-based public health research, where representativeness biases, platform dynamics, and context collapse can affect conclusions [19].

In addition, the counts were raw and nonnormalized. We did not have access to annual denominators, such as total X activity, language-specific posting volume, or active users by language. Therefore, the observed trajectories cannot be interpreted as normalized changes in public interest. Apparent stability in raw mention volumes may also be shaped by algorithmic visibility dynamics, platform rebranding, moderation changes, or shifts in user activity rather than by actual levels of public interest. Relevant discussions may also have migrated to other platforms, such as TikTok (ByteDance), Instagram (Meta Platforms), Reddit, or health-specific online communities. The growing role of “influencers” in the dissemination of TCIM content should also be considered [44].

Because Brandwatch indexes reposts, retweets, and quote posts as mentions, high-volume terms may partly reflect recirculation or amplification of a limited number of posts rather than independent contributions from many users. Moreover, we could not determine whether the users behind the mentions were consumers, patients, caregivers, health professionals, advocates, opponents, organizations, automated accounts, or bots.

The heterogeneity of the TCIM umbrella construct is also a limitation of the aggregate analysis. Because the 39 retained terms refer to different types of practices and vocabularies, they should not be interpreted as equivalent units of TCIM discourse. The lexicon was intentionally restricted to 39 commonly recognized terms. This approach improves feasibility and transparency but does not capture the full anthropological, cultural, doctrinal, and lexical diversity of TCIM-related discourse.

Future studies should examine predefined subcategories and broader lexicons to better distinguish wellness, clinical, traditional, and contested TCIM-related vocabularies.

The multilingual comparison also has limitations. Language was used as a proxy for linguistic space, but it cannot be equated with culture, nationality, or comparable discourse communities. Although lexical variants were considered for selected terms when relevant, translated terms may still not map perfectly onto equivalent semantic uses across English, Spanish, and French.

The exploratory trend analyses were also limited by the small number of annual observations in the 2015 to 2019 window. Even with the exact permutation test, a single year could influence the slope, *R*^2^, and whether a term-language combination met the predefined exploratory criteria. In addition, no multiplicity adjustment was applied across term-language comparisons. These results should therefore be interpreted as hypothesis-generating exploratory trend signals rather than confirmatory evidence of linear change.

Finally, the study reflects only one social media platform. The observed mention patterns may therefore be specific to X and should not be generalized to other digital spaces where TCIM-related discussions may follow different visibility, audience, and dissemination dynamics [45].

### Conclusions

Over the past decade, social media have become an important space for health-related information and discussion. Between 2015 and 2024, raw TCIM-related mention counts on X were highly concentrated in a small set of wellness and body-mind practice labels and were predominantly indexed in English compared with Spanish and French. No sustained aggregate surge was evident in the raw descriptive data during the COVID-19 pandemic period, although specific terms showed short-term fluctuations. These findings should therefore be interpreted as platform-specific raw mention patterns, not as evidence of normalized public interest, sentiment, attitudes, professional practice, or actual TCIM use. Monitoring health-related vocabularies on social media may help inform strategies to improve the dissemination of accurate health information, mitigate misinformation, and strengthen health communication among public and scientific communities [46].

## Appendix

Multimedia Appendix 1 Supplementary material on traditional, complementary, and integrative medicine (TCIM) lexicon construction, AI-assisted term identification, and multilingual search labels.

Multimedia Appendix 2 Annual raw mention counts for the 39 traditional, complementary, and integrative medicine (TCIM)–related terms on X (formerly Twitter), 2015-2024, pooled across English, French, and Spanish.

Multimedia Appendix 3 Supplementary exploratory trend analysis figures for traditional, complementary, and integrative medicine (TCIM)–related mentions on X, 2015-2019.

## Abbreviations

TCIM: traditional, complementary, and integrative medicine
WHO: World Health Organization

## Data Availability

The aggregated data analyzed in this study were obtained through a one-off licensed insights service from Brandwatch Consumer Research (Cision Ltd) and are subject to contractual access restrictions. Brandwatch provided the study team with provider-generated reports and outputs used for the analyses. Therefore, the source data are not publicly available. Requests regarding access should be directed to the data provider and remain subject to the applicable licensing terms and institutional policies.
