# Supplementary material for: Traditional, Complementary, and Integrative Medicine on X (Formerly Twitter) From 2015 to 2024 Across English, Spanish, and French: Retrospective Observational Study
Source: J Med Internet Res. 2026 Jul 27;28:e97760. doi: 10.2196/97760 (PMC13458356; doi:10.2196/97760)
Supplement: Supplementary file 1 [file jmir_v28i1e97760_app1.pdf]

Supplementary material on traditional, complementary, and integrative medicine (TCIM) lexicon construction, AI-assisted term identification, and multilingual search labels.

This is a Multimedia Appendix to a full manuscript published in the J Med Internet Res. For full copyright and citation information see http://dx.doi.org/10.2196/jmir.97760.

Textbox S1. Exact prompt used for AI-assisted triangulation of candidate traditional, complementary, and integrative medicine (TCIM) lexicon terms with ChatGPT, DeepSeek, and Copilot on April 21, 2025.
Context. This prompt was used only for structured brainstorming. The authors subsequently reviewed and adjudicated all AI-generated outputs, which were not considered evidence of clinical relevance, effectiveness, or frequency of use.

You are helping to create a multilingual search lexicon for a retrospective study of public discourse on traditional, complementary, and integrative medicine on X (formerly Twitter).<br>Your task is to identify the practices, approaches, disciplines, and techniques of traditional, complementary, and integrative medicine that are most likely to be frequently mentioned, visible, and regularly discussed on X/Twitter.<br><br>- The goal is not to evaluate clinical efficacy, public health relevance, or real-world use.<br>- The goal is to generate a practical list of potential search terms that can be used in a social media trend analysis.<br><br>Instructions:<br><br>1. Focus on terms that average users are likely to write, mention, search for, use as hashtags, or discuss on X/Twitter.<br><br>2. Prioritize terms that are:<br>- widely recognized in public discourse,<br>- likely to generate a significant volume of mentions,<br>- relevant to traditional, complementary, and integrative medicine.<br><br>3. Cover three semantic levels:<br>- general approaches or systems, - named disciplines or modalities, - specific techniques, methods, or remedies.<br><br>4. Include terms commonly associated with:<br>- well-being, - stress reduction, - natural health, - supportive care, - integrative health, - and spirituality when it clearly overlaps with health-related discourse.<br><br>5. Exclude:<br>- names of people, brands, clinics, or commercial products,<br>- biomedical treatments that are not generally addressed within the context of traditional, complementary, and integrative medicine.<br><br>6. Do not invent data, classifications, frequencies, or references.<br><br>7. If a term seems relevant but uncertain, mark it as uncertain rather than forcing its inclusion.<br><br>8. Keep in mind that the final lexicon will later be translated and used in English, French, and Spanish.<br><br>9. Keep in mind that a term may refer to:<br>- an approach, - a discipline, - a method, - a technique, - or a specific component or remedy.

Expected outcome:

Create a structured table with the following columns:
1. Candidate term
2. Category:
- approach, - discipline, - technique.
3. Why this term is likely to appear in public discourse on X/Twitter

Final output:
At the end, provide a list of the most relevant key terms to include in a final multilingual lexicon.
Important constraints:
- Stay focused on building a lexicon intended for exploring social media discourse on X/Twitter.
- Do not write an essay.
- Do not address the issue of effectiveness.
- Do not discuss regulations in detail, unless it helps explain why a term is likely to circulate publicly.
- Do not interpret the frequency of mentions as actual use by patients.

Table S1. Identification of candidate traditional, complementary, and integrative medicine (TCIM) lexicon entries by ChatGPT, Copilot, and DeepSeek: terms identified by at least 1 AI tool (n=104) and by all 3 AI tools (n=39).

English candidate term identified by AI	ChatGPT	Copilot	DeepSeek
Acupressure	Identified	Identified	Identified
Acupuncture	Identified	Identified	Identified
Anthroposophic medicine	Identified	Identified	Identified
Art therapy	Identified	Identified	Identified
Ayurveda	Identified	Identified	Identified
Buteyko / Buteyko breathing technique	Identified	Identified	Identified
Chiropractic	Identified	Identified	Identified
Complementary medicine	Identified	Identified	Identified
EMDR	Identified	Identified	Identified
Energy healing	Identified	Identified	Identified
Energy medicine	Identified	Identified	Identified
Essential oils	Identified	Identified	Identified
Forest bathing / sylvotherapy	Identified	Identified	Identified
Homeopathy	Identified	Identified	Identified
Hypnotherapy	Identified	Identified	Identified
Integrative medicine	Identified	Identified	Identified
Intermittent fasting	Identified	Identified	Identified
Ketogenic diet	Identified	Identified	Identified
Kinesiology	Identified	Identified	Identified
Meditation	Identified	Identified	Identified
Mindfulness	Identified	Identified	Identified
Moxibustion	Identified	Identified	Identified
Music therapy	Identified	Identified	Identified
Naturopathy	Identified	Identified	Identified
Neurofeedback	Identified	Identified	Identified
Osteopathy	Identified	Identified	Identified
Phytotherapy / herbal medicine	Identified	Identified	Identified
Qi gong / qigong	Identified	Identified	Identified
Reflexology	Identified	Identified	Identified
Reiki	Identified	Identified	Identified
Shamanism	Identified	Identified	Identified
Shiatsu	Identified	Identified	Identified
Sophrology	Identified	Identified	Identified
St John's wort	Identified	Identified	Identified
Tai chi	Identified	Identified	Identified
Thermal therapy	Identified	Identified	Identified
Traditional Chinese medicine	Identified	Identified	Identified
Traditional medicine	Identified	Identified	Identified
Yoga	Identified	Identified	Identified

English candidate term identified by AI	ChatGPT	Copilot	DeepSeek
Alexander technique	Identified	Not identified	Not identified
Aloe vera	Identified	Not identified	Not identified
Alternative medicine	Identified	Not identified	Not identified
Aromatherapy	Identified	Not identified	Not identified
Ashwagandha	Identified	Not identified	Not identified
Auricular acupuncture	Not identified	Not identified	Identified
Ayurvedic massage	Identified	Not identified	Not identified
Balneotherapy	Identified	Not identified	Not identified
Biofeedback	Identified	Not identified	Not identified
Breathwork	Not identified	Identified	Not identified
Cannabis	Not identified	Not identified	Identified
Cardiac coherence	Not identified	Not identified	Identified
Chakra / chakra healing	Identified	Not identified	Not identified
Chamomile	Identified	Not identified	Not identified
Creative visualization	Not identified	Not identified	Identified
Crystal healing / lithotherapy	Identified	Not identified	Not identified
Cupping / hijama	Identified	Not identified	Not identified
Dance therapy	Identified	Not identified	Not identified
Deep breathing	Identified	Not identified	Not identified
Detox / cleanse	Not identified	Not identified	Identified
Dietary supplements	Identified	Not identified	Not identified
Echinacea	Identified	Not identified	Not identified
Elderberry	Identified	Not identified	Not identified
Fish oil / omega-3	Identified	Not identified	Not identified
Functional medicine	Identified	Not identified	Not identified
Garlic	Identified	Not identified	Not identified
Ginger	Identified	Not identified	Not identified
Ginkgo / ginkgo biloba	Identified	Not identified	Not identified
Ginseng	Identified	Not identified	Identified
Green tea	Identified	Not identified	Not identified
Guided imagery	Identified	Not identified	Not identified
Guided meditation	Identified	Identified	Not identified
Healing touch / therapeutic touch	Identified	Not identified	Not identified
Herbal remedies	Identified	Not identified	Identified
Holistic health	Identified	Not identified	Not identified
Hydrotherapy	Identified	Not identified	Identified
Indigenous traditional healing	Identified	Not identified	Not identified
Lavender	Identified	Not identified	Not identified
Massage therapy	Identified	Not identified	Not identified
MBSR	Identified	Not identified	Not identified
Medicinal herbal teas	Identified	Not identified	Not identified
Melatonin	Identified	Not identified	Not identified
Natural health	Identified	Not identified	Not identified

English candidate term identified by AI	ChatGPT	Copilot	DeepSeek
Natural products	Identified	Not identified	Not identified
Peppermint oil	Identified	Not identified	Not identified
Pilates	Identified	Not identified	Not identified
Pranayama / yogic breathing	Identified	Not identified	Not identified
Prayer healing	Identified	Not identified	Not identified
Prebiotics	Identified	Not identified	Not identified
Probiotics	Identified	Not identified	Not identified
Progressive muscle relaxation	Identified	Not identified	Not identified
Relaxation techniques	Identified	Not identified	Not identified
Self-hypnosis	Identified	Not identified	Not identified
Siddha medicine	Identified	Not identified	Not identified
Sound healing / sound bath	Identified	Not identified	Not identified
Spa therapy	Identified	Not identified	Not identified
Spinal manipulation	Identified	Not identified	Not identified
Spiritual healing	Identified	Not identified	Not identified
Tea tree oil	Identified	Not identified	Not identified
Thai massage / Nuad Thai	Identified	Not identified	Not identified
Therapeutic fasting	Not identified	Identified	Not identified
Tuina / Tui na	Identified	Identified	Not identified
Turmeric	Identified	Not identified	Not identified
Unani medicine	Identified	Not identified	Not identified
Valerian	Identified	Not identified	Not identified

Note. AI tool columns indicate whether each candidate term, or a closely equivalent lexical formulation, appeared in the corresponding AI-assisted brainstorming output. The table includes terms identified by at least 1 AI tool (n=104), with terms identified by all 3 AI tools indicated separately (n=39). AI identification was used only for structured brainstorming and should not be interpreted as evidence of clinical relevance, effectiveness, frequency of use, or inclusion in the final search lexicon.

Table S2. Semantic levels, multilingual search labels, and lexical variants for 39 final traditional, complementary, and integrative medicine (TCIM)–related lexicon entries.

Semantic level	English search label	French search label	Spanish search label
Technique	Acupressure	Acupression	Acupresión
Discipline	Acupuncture	Acupuncture	Acupuntura
Approach	Anthroposophic medicine	Médecine anthroposophique	Medicina antroposófica
Discipline	Art therapy	Art-thérapie	Arte terapia
Approach	Ayurveda	Ayurvéda	Ayurveda
Technique	Buteyko or Buteyko breathing technique	Buteyko	Técnica respiratoria Buteyko
Discipline	Chiropractic	Chiropraxie	Quiropráctica
Approach	Complementary medicine	Médecine complémentaire	Medicina complementaria
Technique	EMDR or Eye movement desensitization and reprocessing	EMDR ou Désensibilisation et retraitement par les mouvements oculaires	EMDR o Desensibilización y Reprocesamiento por Movimientos Oculares
Discipline	Energy healing	Soin énergétique ou Soins énergétiques	Sanación energética
Approach	Energy medicine	Médecine énergétique	Medicina energética
Technique	Essential oils	Huiles essentielles	Aceites esenciales
Technique	Forest bathing or sylvotherapy	Sylvothérapie	Silvoterapia
Approach	Homeopathy	Homéopathie	Homeopatía
Discipline	Hypnotherapy	Hypnothérapie	Hipnoterapia
Approach	Integrative medicine	Médecine intégrative	Medicina integrativa
Technique	Intermittent fasting	Jeûne intermittent	Ayuno intermitente
Technique	Ketogenic diet	Cétogène	Dieta cetogénica
Discipline	Kinesiology	Kinésiologie	Kinesiología
Technique	Meditation	Méditation	Meditación
Technique	Mindfulness	Pleine conscience	Atención plena
Technique	Moxibustion	Moxibustion	Moxibustión
Discipline	Music therapy	Musicothérapie	Musicoterapia
Approach	Naturopathy	Naturopathie	Naturopatía
Technique	Neurofeedback	Neurofeedback	Neurofeedback
Discipline	Osteopathy	Ostéopathie	Osteopatía
Discipline	Phytotherapy or herbal medicine	Phytothérapie	Fitoterapia o medicina herbal
Discipline	Qi gong or qigong	Qi gong	Qi gong
Discipline	Reflexology	Réflexologie	Reflexología
Discipline	Reiki	Reiki	Reiki
Approach	Shamanism	Chamanisme	Chamanismo
Discipline	Shiatsu	Shiatsu	Shiatsu
Discipline	Sophrology	Sophrologie	Sofrología
Technique	St John's wort	Millepertuis	Hierba de San Juan
Discipline	Tai chi	Tai-chi	Tai chi

Technique	Thermal therapy	Cure thermale ou cures thermales	Terapia termal o balneoterapia
Approach	Traditional Chinese medicine	Médecine Chinoise ou Médecine traditionnelle Chinoise	Medicina tradicional China
Approach	Traditional medicine	Médecine traditionnelle	Medicina tradicional
Discipline	Yoga	Yoga	Yoga

Note. Each row represents 1 of the 39 final TCIM-related lexicon entries. English, French, and Spanish search labels are shown as used for multilingual term grouping. When multiple lexical variants were grouped under the same entry, they were joined using the language-specific equivalent of "or" and did not increase the number of entries. Cross-language labels should be interpreted as search equivalents and not as evidence of identical semantic, cultural, or discourse uses across languages.
