# Supplementary material for: Traditional, Complementary, and Integrative Medicine on X (Formerly Twitter) From 2015 to 2024 Across English, Spanish, and French: Retrospective Observational Study
Source: J Med Internet Res. 2026 Jul 27;28:e97760. doi: 10.2196/97760 (PMC13458356; doi:10.2196/97760)
Supplement: Supplementary file 2 [file jmir_v28i1e97760_app2.pdf]

Annual raw mention counts for the 39 traditional, complementary, and integrative medicine (TCIM)–related terms on X (formerly Twitter), 2015-2024, pooled across English, French, and Spanish.

This is a Multimedia Appendix to a full manuscript published in the J Med Internet Res. For full copyright and citation information see http://dx.doi.org/10.2196/jmir.97760.

Table S3. Annual raw mention counts for 39 traditional, complementary, and integrative medicine (TCIM)–related terms on X, 2015-2024, pooled across English, French, and Spanish.

TCIM–related lexicon entry	2015	2016	2017	2018	2019	2020	2021	2022	2023	2024
Yoga	15,659,632	12,244,782	10,035,405	7,355,588	5,635,251	7,061,449	5,421,368	5,866,108	5,141,235	5,197,365
Meditation	6,822,878	6,367,315	5,320,873	4,285,088	3,627,557	5,563,506	5,572,602	7,002,325	6,803,044	4,195,184
Mindfulness	1,897,349	2,727,219	3,328,490	2,575,704	1,594,420	1,689,951	1,521,444	1,416,072	1,142,738	1,021,990
Ayurveda	354,801	324,952	312,763	311,305	214,901	615,525	773,696	539,272	453,611	495,904
Essential oils	570,609	516,652	560,608	580,125	394,366	386,412	297,824	190,414	159,450	132,201
Homeopathy	378,671	428,548	543,992	691,708	465,008	332,789	225,178	205,664	179,390	153,688
Reiki	770,241	558,754	483,965	326,653	240,833	267,617	208,457	194,023	157,880	191,702
Chiropractic	527,061	660,762	656,102	422,179	258,950	198,240	194,410	178,681	136,500	148,275
Tai chi	463,622	564,148	443,191	367,327	372,593	308,083	184,905	200,817	213,663	212,159
Acupuncture	638,601	411,425	363,646	302,901	217,926	205,479	164,049	180,685	149,950	128,906
Intermittent fasting	73,358	71,376	95,675	219,341	266,022	342,453	250,633	312,950	383,248	281,254
Traditional medicine	55,282	46,631	48,728	67,074	79,882	150,706	98,536	117,369	104,156	102,613
Hypnotherapy	213,092	158,799	107,735	104,769	66,251	50,749	47,886	44,939	36,606	33,477
Music therapy	98,885	99,836	95,077	132,193	85,221	84,052	66,155	77,667	57,062	51,438
Ketogenic diet	43,383	60,852	130,067	184,627	95,701	79,869	57,091	58,413	57,897	68,674
Kinesiology	146,578	87,623	80,854	80,529	123,285	75,530	68,539	71,595	49,501	47,819
Reflexology	178,955	132,838	94,958	70,228	49,776	33,668	27,831	38,344	25,421	20,523
Art therapy	71,470	65,521	89,753	64,576	50,479	54,055	50,715	47,368	40,876	39,542
Acupressure	62,452	55,987	70,710	47,415	34,600	56,755	35,015	110,884	46,452	51,205
Phytotherapy	55,396	43,932	48,508	48,479	39,949	71,134	49,838	55,991	78,177	46,179

TCIM–related lexicon entry	2015	2016	2017	2018	2019	2020	2021	2022	2023	2024
Qigong	96,192	68,745	50,560	50,993	38,192	38,116	34,137	40,157	39,301	32,910
Shiatsu	115,778	89,368	64,689	46,113	25,102	24,837	23,323	23,034	17,225	12,455
Traditional Chinese medicine	31,806	29,153	30,578	48,031	61,328	79,792	30,361	41,627	38,794	37,749
Osteopathy	66,320	59,292	49,035	48,175	41,408	31,204	29,204	31,779	31,765	39,522
Naturopathy	36,739	39,043	36,050	37,455	32,610	65,784	39,456	47,763	47,626	39,473
Shamanism	29,339	28,511	36,359	35,422	26,764	27,053	30,247	35,470	33,934	85,508
Energy healing	44,232	50,255	54,810	35,545	29,979	28,765	26,132	25,689	25,764	29,911
EMDR	14,624	16,029	21,646	29,047	27,530	36,907	49,072	35,658	31,455	29,033
Sophrology	20,987	38,575	43,180	25,823	27,926	23,009	19,279	18,938	12,078	6,733
Integrative medicine	22,169	18,938	21,794	20,081	16,825	16,505	17,178	20,289	21,479	17,548
Forest bathing	4,105	8,940	29,880	27,465	19,565	18,495	15,903	19,785	14,592	13,864
Neurofeedback	20,139	19,209	21,721	16,788	14,514	12,446	18,221	14,179	11,887	9,430
Energy medicine	33,260	14,375	7,574	6,939	5,518	8,172	4,028	5,624	6,596	6,152
Complementary medicine	14,935	12,014	10,028	12,031	8,014	4,223	4,262	6,458	4,770	2,882
St John's wort	7,923	8,184	7,504	9,191	5,622	6,982	5,451	5,190	4,944	4,812
Moxibustion	11,414	6,461	4,479	3,711	3,606	3,619	2,939	3,500	2,841	3,472
Thermal therapy	1,856	2,202	2,402	2,465	3,586	2,498	3,254	2,670	3,656	5,463
Buteyko breathing technique	2,163	1,911	2,296	1,827	1,508	1,602	1,117	952	1,140	2,214
Anthroposophic medicine	502	351	345	438	1,836	1,651	4,405	2,260	1,354	1,045
**Total**	29,656,799	26,139,508	23,406,030	18,695,349	14,304,404	18,059,682	15,674,141	17,290,603	15,768,058	13,000,274

Note. Values are raw aggregated mention counts pooled across English, French, and Spanish and were not normalized by total X activity or language-specific posting volume. Lexical variants grouped under the same entry were aggregated. Counts represent platform-specific mention volumes and should not be interpreted as measures of TCIM use, endorsement, sentiment, or unique users.
