# Supplementary material for: Traditional, Complementary, and Integrative Medicine on X (Formerly Twitter) From 2015 to 2024 Across English, Spanish, and French: Retrospective Observational Study
Source: J Med Internet Res. 2026 Jul 27;28:e97760. doi: 10.2196/97760 (PMC13458356; doi:10.2196/97760)
Supplement: Supplementary file 3 [file jmir_v28i1e97760_app3.pdf]

Supplementary exploratory trend analysis figures for traditional, complementary, and integrative medicine (TCIM)–related mentions on X, 2015-2019.

This is a Multimedia Appendix to a full manuscript published in the J Med Internet Res. For full copyright and citation information see http://dx.doi.org/10.2196/jmir.97760.

Figure S1. Relative change in raw mention counts from 2015 to 2019 for English-language traditional, complementary, and integrative medicine (TCIM)–related terms on X meeting the predefined exploratory criteria ($R^2 \geq 0.70$ and $P \leq .05$; n=14). Error bars indicate 95% CIs, and bar shading represents the 2015 raw mention volume on a logarithmic scale.

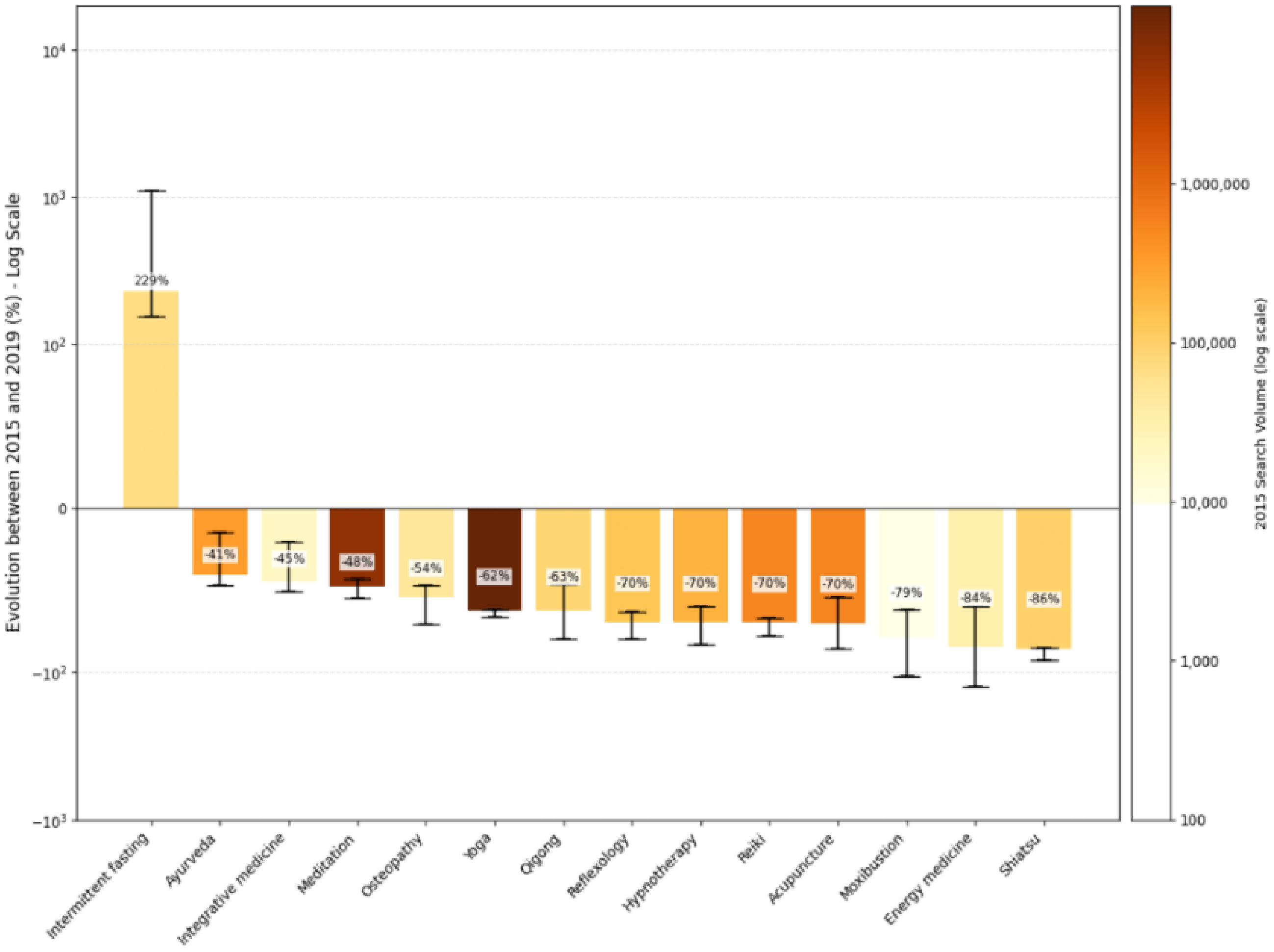

Figure S2. Relative change in raw mention counts from 2015 to 2019 for French-language traditional, complementary, and integrative medicine (TCIM)–related terms on X meeting the predefined exploratory criteria ($R^2 \geq 0.70$ and $P \leq .05$; n=11). Error bars indicate 95% CIs, and bar shading represents the 2015 raw mention volume on a logarithmic scale.

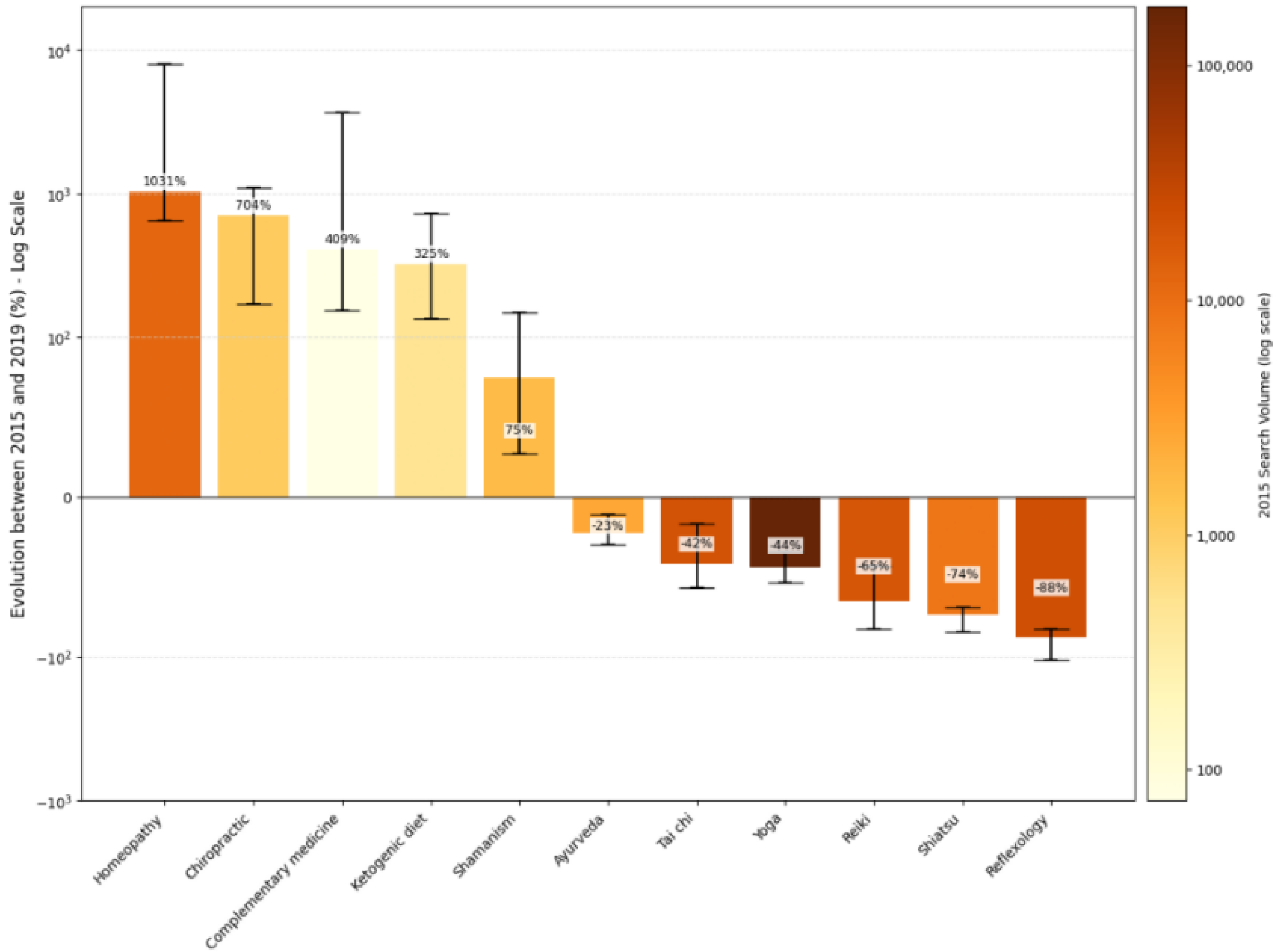

Figure S3. Relative change in raw mention counts from 2015 to 2019 for Spanish-language traditional, complementary, and integrative medicine (TCIM)–related terms on X meeting the predefined exploratory criteria ($R^2 \geq 0.70$ and $P \leq .05$; n=8). Error bars indicate 95% CIs, and bar shading represents the 2015 raw mention volume on a logarithmic scale.

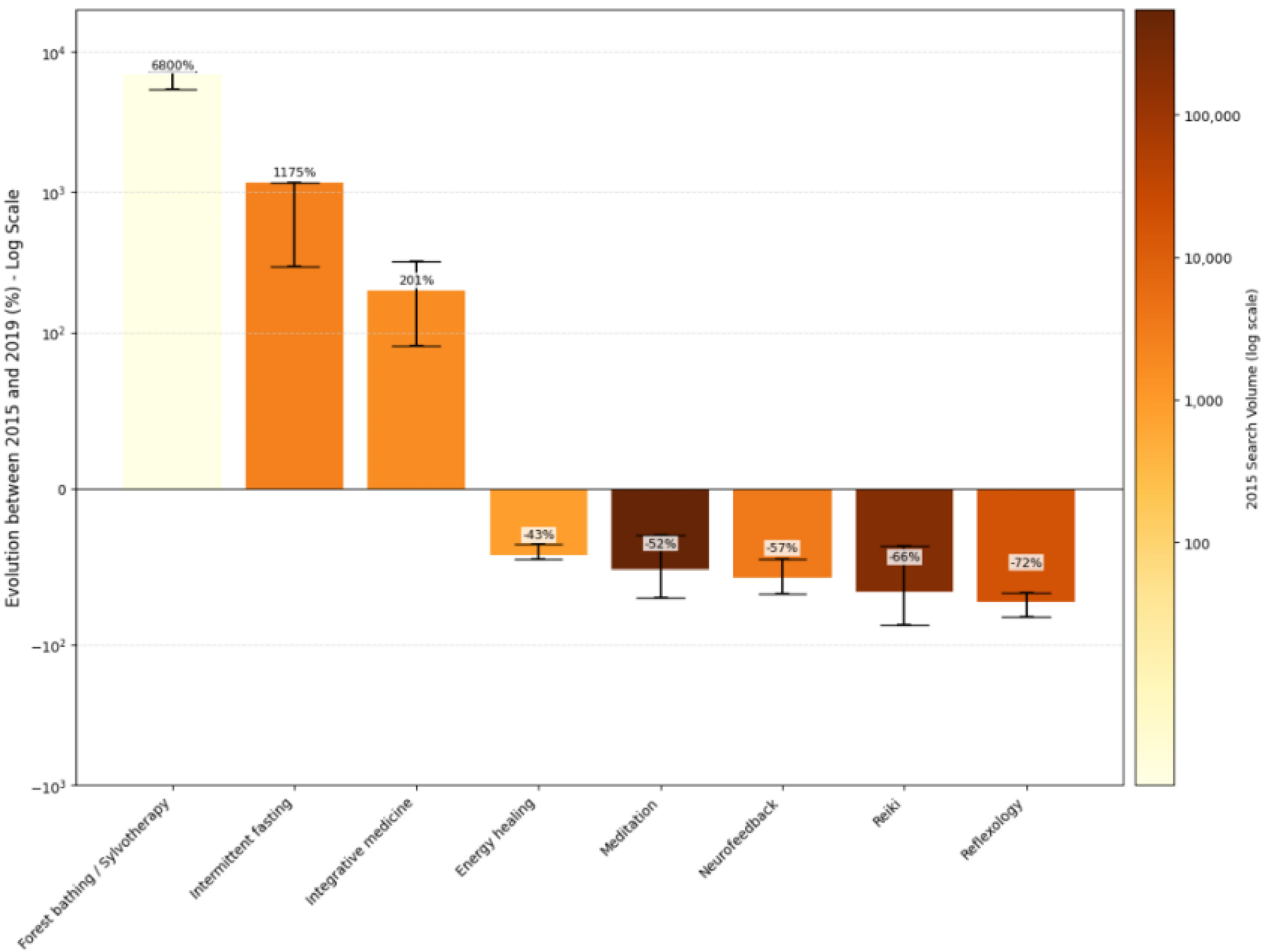

Figure S4. Exploratory trend-analysis results for 39 traditional, complementary, and integrative medicine (TCIM)–related terms on X by language, 2015 to 2019. For each term-language combination, the figure reports R^2, the P value from the exact permutation test on the slope, the estimated annual slope, the 2015 raw mention count, and whether both predefined criteria ($R^2 \geq 0.70$ and $P \leq .05$) were met. Overall, 14 English-language, 11 French-language, and 8 Spanish-language combinations met the criteria.

French-language analysis—11 terms met the predefined criteria

Term	R^2	P value	Slope	2015 value	Meets criteria
Shiatsu	0.967	.02	−1694.000	8 479	✓ Yes
Reflexology	0.945	.02	−5676.800	23 094	✓ Yes
Yoga	0.929	.02	−19,738.000	177 612	✓ Yes
Ketogenic diet	0.908	.02	432.800	495	✓ Yes
Complementary medicine	0.884	.03	70.500	74	✓ Yes
Homeopathy	0.848	.02	33,281.800	11 925	✓ Yes
Chiropractic	0.818	.05	2454.700	1 098	✓ Yes
Reiki	0.803	.05	−3629.100	18 801	✓ Yes
Ayurveda	0.795	.05	−144.500	2 638	✓ Yes
Intermittent fasting	0.777	.10	418.300	342	× No
Shamanism	0.755	.05	281.000	1 674	✓ Yes
Tai chi	0.743	.03	−1804.300	20 750	✓ Yes
Integrative medicine	0.729	.08	112.700	106	× No
St John's wort	0.693	.10	62.700	598	× No
Naturopathy	0.675	.08	710.200	3 712	× No
EMDR	0.673	.05	224.100	802	× No
Thermal therapy	0.567	.10	301.100	1 394	× No
Art therapy	0.543	.15	−472.300	3 458	× No
Anthroposophic medicine	0.530	.07	299.300	8	× No
Kinesiology	0.508	.15	−805.300	2 532	× No
Acupressure	0.470	.22	−454.000	2 970	× No
Traditional Chinese medicine	0.451	.20	1417.400	2 230	× No
Energy healing	0.444	.22	−382.000	1 495	× No
Acupuncture	0.412	.22	436.400	5 763	× No
Forest bathing or sylvotherapy	0.345	.32	311.900	44	× No
Traditional medicine	0.332	.32	345.600	4 158	× No
Moxibustion	0.252	.38	8.700	59	× No
Mindfulness	0.235	.42	1416.000	10 203	× No
Herbal medicine or phytotherapy	0.233	.42	132.300	3 475	× No
Hypnotherapy	0.173	.47	−36.900	888	× No
Qigong	0.145	.52	−132.800	2 742	× No
Energy medicine	0.135	.60	14.100	62	× No
Buteyko breathing technique	0.029	.82	0.500	2	× No
Osteopathy	0.021	.85	−100.500	8 911	× No
Meditation	0.013	.87	2088.600	107 853	× No
Neurofeedback	0.010	.90	−3.800	600	× No
Music therapy	0.001	>.99	2.300	1 449	× No
Essential oils	0.000	.98	100.800	20 072	× No
Sophrology	0.000	>.99	−28.100	19 804	× No

English-language analysis—14 terms met the predefined criteria

Term	R^2	P value	Slope	2015 value	Meets criteria
Yoga	0.997	.02	−2,113,203.100	13,265,285	✓ Yes
Shiatsu	0.993	.02	−21,331.000	98,744	✓ Yes
Reiki	0.984	.02	−95,214.400	536,832	✓ Yes
Meditation	0.979	.02	−786,175.500	6,170,769	✓ Yes
Reflexology	0.961	.02	−23,404.300	138,766	✓ Yes
Hypnotherapy	0.939	.02	−34,822.200	209,612	✓ Yes
Osteopathy	0.921	.02	−6909.000	48,170	✓ Yes
Acupuncture	0.895	.02	−89,451.900	559,364	✓ Yes
Qigong	0.868	.03	−12,733.000	87,837	✓ Yes
Intermittent fasting	0.838	.03	46,095.500	70,496	✓ Yes
Moxibustion	0.808	.02	−1772.900	9629	✓ Yes
Ayurveda	0.757	.03	−27,278.300	330,255	✓ Yes
Integrative medicine	0.756	.05	−1827.700	20,383	✓ Yes
EMDR	0.753	.07	3148.800	12,020	× No
Energy medicine	0.733	.02	−6246.800	32,936	✓ Yes
Naturopathy	0.706	.07	−2445.100	29,452	× No
Traditional medicine	0.674	.12	3491.500	20,652	× No
Essential oils	0.603	.13	−33,757.700	534,663	× No
Complementary medicine	0.601	.13	−1100.100	10,201	× No
Traditional Chinese medicine	0.600	.13	2266.700	21,296	× No
Buteyko breathing technique	0.581	.15	−171.000	2082	× No
Homeopathy	0.572	.12	−21,986.500	272,320	× No
Chiropractic	0.539	.15	−80,320.900	519,307	× No
Acupressure	0.506	.20	−5722.400	57,271	× No
Tai chi	0.491	.17	−34,877.200	415,698	× No
Forest bathing or sylvotherapy	0.457	.22	4596.700	4059	× No
Herbal medicine or phytotherapy	0.433	.22	−2140.900	43,209	× No
Kinesiology	0.430	.25	−11,790.900	118,687	× No
Energy healing	0.404	.27	−3854.100	41,875	× No
Ketogenic diet	0.401	.25	19,336.900	40,421	× No
Neurofeedback	0.398	.25	−849.000	16,062	× No
Sophrology	0.390	.23	132.700	940	× No
Thermal therapy	0.335	.28	71.900	436	× No
St John's wort	0.314	.35	−437.000	6067	× No
Art therapy	0.146	.58	−3302.800	64,699	× No
Mindfulness	0.038	.75	−82,308.000	1,812,309	× No
Music therapy	0.029	.85	1719.100	73,563	× No
Shamanism	0.006	.85	−224.700	22,160	× No
Anthroposophic medicine	0.004	.93	−3.100	250	× No

Spanish-language analysis—8 terms met the predefined criteria

Term	R^2	P value	Slope	2015 value	Meets criteria
Energy healing	0.973	.02	−85.500	862	✓ Yes
Reflexology	0.967	.02	−3015.700	17,095	✓ Yes
Forest bathing or sylvotherapy	0.944	.02	35.900	2	✓ Yes
Neurofeedback	0.943	.02	−514.300	3477	✓ Yes
Integrative medicine	0.910	.03	760.500	1680	✓ Yes
Meditation	0.809	.02	−63,200.000	544,256	✓ Yes
Intermittent fasting	0.788	.02	6815.500	2520	✓ Yes
Reiki	0.786	.02	−30,248.200	214,608	✓ Yes
Art therapy	0.704	.08	−517.600	3313	× No
Naturopathy	0.687	.02	750.300	3575	× No
Qigong	0.674	.03	−509.400	5613	× No
Yoga	0.674	.02	−360,854.500	2,216,735	× No
Acupuncture	0.655	.10	−5971.900	73,474	× No
EMDR	0.624	.10	510.100	1802	× No
Kinesiology	0.607	.10	7228.200	25,359	× No
Traditional Chinese medicine	0.527	.17	4108.100	8280	× No
Traditional medicine	0.400	.23	3127.200	30,472	× No
Anthroposophic medicine	0.380	.25	−20.700	244	× No
Ayurveda	0.352	.28	−1921.900	21,908	× No
Moxibustion	0.345	.30	−72.400	1726	× No
Shamanism	0.336	.32	119.800	5505	× No
Ketogenic diet	0.335	.30	3071.400	2467	× No
Herbal medicine or phytotherapy	0.317	.37	−626.100	8712	× No
Sophrology	0.315	.33	8.000	243	× No
Osteopathy	0.307	.30	915.400	9239	× No
Chiropractic	0.293	.33	385.700	6656	× No
Homeopathy	0.290	.37	32,288.100	94,426	× No
Music therapy	0.286	.43	−1218.500	23,873	× No
Buteyko breathing technique	0.276	.42	31.100	79	× No
Tai chi	0.236	.47	−1206.400	27,174	× No
Shiatsu	0.234	.43	564.300	8555	× No
Acupressure	0.213	.43	−251.200	2211	× No
Essential oils	0.209	.42	4755.600	15,874	× No
Energy medicine	0.204	.50	−59.300	262	× No
Mindfulness	0.191	.48	5154.700	74,837	× No
Complementary medicine	0.188	.52	−352.900	4660	× No
Thermal therapy	0.034	.82	−0.700	26	× No
Hypnotherapy	0.013	.90	87.900	2592	× No
St John's wort	0.005	.83	14.800	1258	× No
